# Under-Prescription of Drugs in the Elderly Population of Western Romania: An Analysis Based on STOPP/START Version 2 Criteria

**DOI:** 10.3390/jcm13195970

**Published:** 2024-10-08

**Authors:** Petru Baneu, Andreea Prelipcean, Valentina Oana Buda, Narcisa Jianu, Anca Tudor, Minodora Andor, Cristina Merlan, Mirabela Romanescu, Maria Suciu, Simona Buda, Teodora Mateoc, Daniela Gurgus, Liana Dehelean

**Affiliations:** 1 Doctoral School, “Victor Babeş” University of Medicine and Pharmacy, 2 Eftimie Murgu Street, 300041 Timisoara, Romania; petru.baneu@umft.ro (P.B.); narcisa.dinu@umft.ro (N.J.); cristina.merlan@umft.ro (C.M.); mirabela.romanescu@umft.ro (M.R.); teodora.mateoc-sirb@umft.ro (T.M.); 2 Faculty of Medicine, “Victor Babeş” University of Medicine and Pharmacy, 2 Eftimie Murgu Street, 300041 Timisoara, Romania; atudor@umft.ro (A.T.); andor.minodora@umft.ro (M.A.); gurgus.daniela@umft.ro (D.G.); lianadeh@umft.ro (L.D.); 3 Institute of Cardiovascular Diseases Timisoara, 13A Gheorghe Adam Street, 300310 Timisoara, Romania; 4 Research Center for Pharmaco-Toxicological Evaluation, “Victor Babeș” University of Medicine and Pharmacy, Eftimie Murgu Sq. no. 2, 300041 Timisoara, Romania; suciu.maria@umft.ro; 5 Faculty of Pharmacy, “Victor Babeș” University of Medicine and Pharmacy, Eftimie Murgu Sq. no. 2, 300041 Timisoara, Romania; 6 Gina Farm Community Pharmacy, Decebal Street no. 2A, 315300 Ineu, Romania; 7 Department of Balneology, Medical Recovery and Rheumatology, Family Discipline, Center for Preventive Medicine, “Victor Babeș” University of Medicine and Pharmacy, 300041 Timisoara, Romania

**Keywords:** inappropriate prescribing, medication review, Romanian healthcare policies, underuse, statins, beta-blockers, antiresorptive therapy

## Abstract

**Background/Objectives**: Numerous European countries, including Romania, are facing the concern of rapid ageing of their populations. Moreover, Romania’s life expectancy ranks among the lowest in the European Union. In light of this, it is imperative that the assessment of medication-related harm be given national priority in order to secure and enhance pharmacotherapy and the medical act. In this study, we sought to describe and evaluate the under-prescribing practices among the Romanian elderly population. **Methods**: We conducted a cross-sectional study in urban areas of two counties in Western Romania (Timis and Arad) from November 2017 to February 2019. We collected chronic electronic prescriptions issued for elderly patients (>65 years old) with chronic conditions. The medication was prescribed by generalist or specialist physicians for periods ranging between 30 and 90 days. To assess inappropriate prescribing behaviours, a multidisciplinary team of specialists applied the Screening Tool of Older Persons’ Prescriptions/Screening Tool to Alert to Right Treatment (STOPP/START) v.2 criteria to the collected prescriptions. **Results**: Within the 1498 prescriptions included in the study, 57% were issued to females, the mean age was 74.1 ± 6.95, and the average number of medicines per prescription was 4.7 ± 1.51. The STOPP criteria most commonly identified were the (1) long treatment duration (23.6%) and (2) prescription of neuroleptics (14.6%) or zopiclone (14.0%) as medications that increase the risk of falls. According to START criteria, the following medicines were under-prescribed: (1) statins (47.4%), (2) beta-blockers (24.5%), (3) antiresorptive therapy (10.0%), and (4) β2-agonists and muscarinic antagonists for chronic obstructive pulmonary disease (COPD) (4.5%). Within our study group, the prevalence of potentially inappropriate medications was 18.58%, whereas the prevalence of potential prescribing omissions was 49.2%. **Conclusions**: To decrease medication-related harm and morbid-mortality, and to increase the quality of life for elderly people in Romania, immediate actions are needed from national authorities. These actions include reinforcing primary care services, providing periodic training for physicians, implementing medication review services by pharmacists, and utilising electronic health records at their full capacity.

## 1. Introduction

Population ageing is a global phenomenon characterised by an increase in both the proportion and size of the elderly population [1,2,3], with serious implications on many aspects of society and the economy including healthcare, social protection, housing, labour markets, and family structures [2,3]. While in high-income countries, such as Japan, population ageing is an already well-established process, other countries, such as Saudi Arabia and South Africa, continue to have predominantly young populations. In Europe, the demographics of ageing populations have been noticeable for decades now and are projected to persist; the main drivers are increased longevity, declining natality rate, and migration patterns [1,3].

Romania is also expected to face a steeper shift in the distribution of the population towards older ages. According to the 2022 Eurostat data, 19.5% of the Romanian population was over the age of 65, whilst the European proportion was 21.1%. Not only is the ratio of aged individuals higher than that presented in the 2017 report (17.8%) but it is also predicted to rise dramatically by 2050 (31%) [4,5]. The ageing population trend in Romania is in line with the steady rise in the number of elderly Europeans, which is projected to attain 29.4% over the course of the next three decades [3]. However, as opposed to the life expectancy of Europeans (80.7 years), people living in Romania have shorter lifespans (75.3 years). Although the life expectancy of Romanians has increased by approximately 4 years since 2000 (71.2 years), the rise remains among the lowest in the European Union (EU) rankings [4,5].

It is broadly acknowledged that the ageing process is associated with a higher incidence of chronic pathologies, which can cause older individuals to lose their independence and require assistance to carry out daily tasks such as eating, dressing, or bathing [3]. Given their longer lifespan, women are more likely to live alone, to become frail, and to require assistance. Senior females also face greater constraints in their day-to-day activities than their male counterparts, with around 25% of women reporting severe difficulties in walking, compared to 15.3% of men. In Romania, the proportion of women over 65 with multiple chronic pathologies is comparable to the EU’s average; the most prevalent conditions are arthrosis, back and neck problems, depression, and hypertension [3,5,6]. However, while approximately one-fifth of European women aged 75 years or older benefit from homecare services, less than one in ten Romanian women in the same age group receive such assistance [3].

According to Eurostat, 4.9% of Romanian people report unmet healthcare needs, compared to a much lower European average of 2.2%. The main reasons include high costs and impaired access to physicians [5]. The Romanian healthcare system is currently based mostly on inpatient care rather than outpatient care and is characterised by major differences regarding access to healthcare between urban and rural areas [5,7]. Around 91% of the hospitals and 92% of outpatient clinics are located in urban areas [7], making it difficult for people living in rural areas to access medical services when they need them, as they face the issue of travelling greater distances, which also increases costs. Regarding the long-term care of elderly people, further improvements are needed, as only 0.23% of people receive home care services. The Romanian system relies mostly on family to provide long-term care for their relatives or on unpaid caregivers [8]. The informal and family-based care model of elderly people is similar to other Eastern European countries, such as Bulgaria, Slovenia and Slovakia. This model has a disadvantage in that it relies often on caregivers who are not properly trained to attend to elderly people and their complex needs [9,10,11].

**Figure 1:**
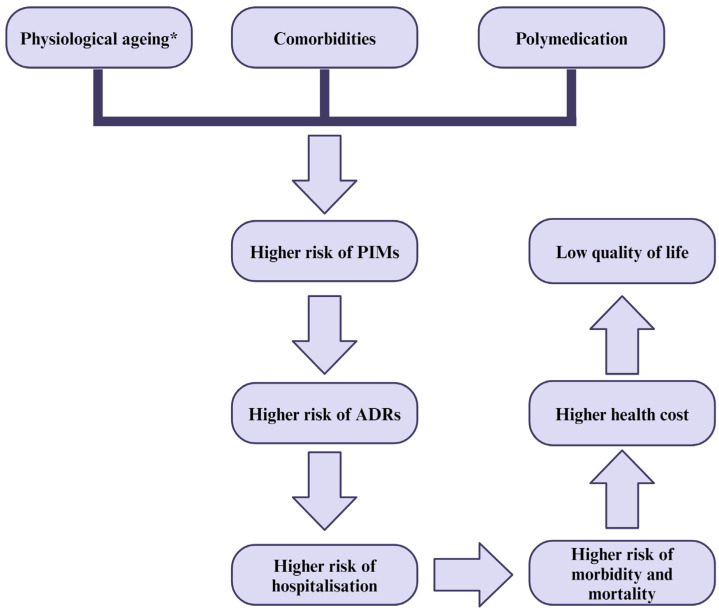
Factors that influence the quality of life of elderly people. * Note: which will induce pharmacokinetic and pharmacodynamic changes.

Initiating and monitoring treatment in elderly patients presents significant challenges due to age-related physiological changes, which have repercussions on the pharmacokinetics and pharmacodynamics of drugs. Additionally, the presence of comorbidities and polypharmacy (defined as the concurrent use of five or more medicines) further complicate treatment management [12,13,14]. Due to the complex treatment regimens typically required, this particular category of patients is at a higher risk of drug interactions and potentially inappropriate medications (PIMs), which result in higher hospitalisation and mortality rates (Figure 1) [12,13,15]. Remarkably, medication review stands out as one of the most efficient tools for promoting medication safety [16] and reducing adverse drug reactions (of which two-thirds are considered to be preventable) in elderly patients [17]. However, whether undertaken by physicians, pharmacists or geriatricians, optimising drug prescribing remains an ongoing challenge. The matter is also acknowledged by the World Health Organization (WHO) in its “Medication without harm” campaign, which tackles the shortcomings of polypharmacy [17]. According to the 2024 WHO report on preventable medication-related harm in healthcare, one in twenty patients is currently negatively impacted by therapy, with 25% of the treatment repercussions being severe or life-threatening; notably, geriatric patients are at higher risk [18]. Addressing the burden of medication-related harm, as outlined in the WHO’s third Global Patient Safety Challenge, is critically needed in an ageing society. In addition to enhancing patients’ quality of life, the benefits encompass lowering the hospitalisation and re-hospitalisation rates, iatrogenic morbid-mortality, and healthcare expenditure. This desideratum could be accomplished by means of patient-centred therapy, multi- and interdisciplinary collaboration among medical professionals, innovative healthcare approaches, and improvements to patients’ therapeutic education through adequate guidance, supervision, and follow-up [19,20].

STOPP (Screening Tool of Older Persons’ Prescriptions) and START (Screening Tool to Alert to Right Treatment) criteria were the pioneering European tools in terms of medication management and assessment [12,13]. Explicitly designed to streamline the medication review process for elderly people in clinical practice, the two tools assess both PIMs and potential prescribing omissions (PPOs) [12,21,22]. PIMs refer to either the unjustified use of drugs—when there is no clear indication (over-prescribing or ”overuse”)—or the inappropriate use of drugs, when, although medication is needed, the risks outweigh the benefits and safer and more effective alternatives are available (mis-prescribing or ”misuse”). PPOs refer to insufficient use of drugs—when beneficial medicines are not prescribed, despite a clear indication (under-prescribing or ”underuse”) [23,24,25]. Applying STOPP/START criteria is feasible and, as opposed to traditional medication review, can be considered simple and time-effective; however, it requires functional and updated software and specialised personnel—a clinical pharmacist who can explain and adapt STOPP/START recommendations to the given clinical context, collaborating with the attending physician [21,26]. Thus, the input of a clinical pharmacist can assist in addressing one of the biggest challenges faced by clinicians: navigating the complex pharmacotherapy of elderlies, without creating suboptimal prescribing problems.

The present study aims to identify and discuss PPOs—namely the under-prescribing of medicines—in the Romanian elderly population based on the country’s state of health profile. This work builds upon the efforts of our research group, which has previously conducted two other studies addressing the issue of inappropriate prescribing in the Western part of Romania, encompassing both urban and rural areas [27,28].

## 2. Materials and Methods

### 2.1. Study Design

A cross-sectional study comprising a number of 1498 electronic medical prescriptions for chronic conditions (EMPCCs) was conducted over a 16-month period, from November 2017 to February 2019. The EMPCCs were collected from 15 randomly elected community pharmacies which agreed to participate in the study (23 pharmacies were initially invited to collaborate), located in the urban areas of two Western Romanian counties: Timis (TM) and Arad (AR) counties. The study area was chosen based on (a) several differences between the two counties (to name a few, the number of physicians in clinical specialties in a contractual relationship with NHIH specialised ambulatory healthcare was 338 physicians in AR County versus 1235 physicians in TM County as reported in 2021 [29]; moreover, TM County has a prestigious medical university and a number of medical research centres), (b) the study group of pharmacists working in these areas, and (c) the pharmacies’ agreement to participate. According to Romanian legislation, the minimum indicators for an urban area are 5000 inhabitants per locality, 75% of the population employed in non-agricultural activities, 70% of all houses equipped with water supply systems, 55% of all dwellings equipped with a bathroom and toilet inside the house, and 7 hospital beds and 1.8 physicians per 1000 inhabitants [30].

Prior to the study debut, a group meeting was held to decide upon the most important study design aspects, including the prescription collection source, study period, selection criteria and methodology. Firstly, the study protocol was drawn up by a group of pharmacists (clinical pharmacists, general practice pharmacists, clinical pharmacy residents, and pharmacy students), in close collaboration with a multidisciplinary team of specialists (cardiologist, internist, psychiatrist, neurologist, rheumatologist, pulmonologist, gastroenterologist, and general practitioner). The group of pharmacists then made contact with a number of pharmacies within the two counties to request an agreement to collaborate. Each partner pharmacy has delegated 2 pharmacists who, aware of the selection criteria, had the responsibility of gathering the EMPCCs and securing personal data by blurring the patients’ personal insurance code (CID). The National Health Insurance System’s diagnostic codes allowed for a unique identification of the patients’ chronic illnesses. The EMPCCs were collected monthly by pharmacy students.

There are a number of Romanian regulatory aspects that are relevant to the present study. According to National Health Insurance House (NHIH) legislation, a chronic electronic prescription is allowed to have a maximum of 7 active substances. The actives are usually prescribed under the international non-proprietary name or, in certain medically justified cases, such as biological products, under their trade name [31]. Romania’s health system operates under a primary health care law, as part of the Law no. 95/2006 on health care reform, which was amended in 2020 to include telemedicine. The social health insurance system is under the governance of the Ministry of Health and NHIH [7]. NHIH is a public institution whose objective is to ensure the efficient functioning of the social health insurance system in Romania by managing the collected funds and the provision of the medical services required by the insured. Thus, insured individuals benefit from access to treatment through a prescription for medicines that are mentioned in the list formulated by the Ministry of Health and NHIH, which are part of the settlement system [7].

### 2.2. Selection Criteria

#### 2.2.1. Inclusion Criteria

We only selected the EMPCCs that were intended for patients aged 65 or older. Further, we included in the analysis the EMPCCs with active ingredients that simultaneously met the following criteria: (1) were eligible for NHIH reimbursement; (2) were intended for ambulatory treatment; and (3) were prescribed for chronic treatment, as a consequence of a chronic diagnosis established by the physician. Hereby, chronic treatment denotes medical therapy administered for more than 3 months to manage a health condition that can typically be controlled but not cured [32].

#### 2.2.2. Exclusion Criteria

We restricted our analysis to electronic medical prescriptions containing active substances which (1) were intended for acute or subacute pathologies; (2) were part of other national programmes requiring distinct EMPCC forms, such as diabetes, oncological diseases, and hepatitis; (3) represented over-the-counter (OTC) medication and dietary supplements; and (4) were categorised as narcotic and psychotropic drugs. According to Romanian legislation, Law 339/2005, the prescription and issuance of narcotic and psychotropic drugs are subject to stricter regulations, requiring a non-electronic, secured prescription form [33]. Also, duplicate prescriptions—EMPCCs containing the same active components, issued for the same patient, but in different months—were not included in the present study.

### 2.3. Data Curation and Assessment

The collected EMPCCs were divided into blocks of 250 prescriptions and subsequently curated. During weekly face-to-face meetings, the interdisciplinary team of specialists evaluated the prescriptions based on STOPP/START v. 2 criteria [22]. Out of the total number, only 26 STOPP and 12 START criteria [22,28] were applicable to the current study, due to the lack of access to patient‘s clinical data and the absence of additional data on pathology characteristics.

The analysis of the prescription forms allowed the team to identify the following data: (1) age; (2) sex; (3) treatment type—ambulatory and chronic; (4) prescribing doctor—general practitioners or specialist physicians; and (5) treatment duration, based on the number of days for which the prescription was issued—30, 60, or 90 days. To increase the accuracy of the screening process, EMPCCs were evaluated in 3 stages. First, the panel of pharmacists evaluated the prescriptions; thereafter, the second assessment was carried out by a general practitioner and a physician with dual specialisation—cardiology and internal medicine. Any encountered problems or unanswered questions were solved in the final stage by the remaining specialists in the team: cardiologist, psychiatrist, neurologist, rheumatologist, pulmonologist, and gastroenterologist. The final report was drawn up on the basis of a unanimous decision of the research group. The present methodology of the EMPCCs’ data evaluation is presented in a previous study published by our group [27]. Lastly, to quantify inappropriate drug recommendations and prescribing errors, we also assessed the prevalence of EMPCCs’ diagnosis codes and analysed drug leaflets. We also sought to identify any differences in terms of prescribing practices between the two counties.

No data were missing from the EMPCCs as the NHIH software used for writing medical prescriptions does not allow for final validation unless the physician completes all the required fields on the EMPCC.

The collected EMPCCs are currently stored securely under lock at the “Victor Babes” University of Medicine and Pharmacy, along with the flash drive containing the analysed electronic data.

### 2.4. Data Analysis

The Statistical Package for Social Sciences (SPSS, version 17.0. Chicago: SPSS Inc., Chicago, IL, USA) software was used to analyse the data recorded in Microsoft Excel.

The study variables are nominal variables (represented by absolute and relative frequencies): gender, the environment of EMPCC provenience, physician, days of treatment (30, 60, or 90), diagnostic (code—description), START/ STOPP criteria, and prescription errors; and numerical variables (represented by mean and standard deviation): age and number of prescribed drugs.

Qualitative variables were described as percentages, while quantitative variables were described as mean ± standard deviation (SD). The Mann–Whitney test was used to observe differences between study groups, and the chi-square test was used to analyse categorical data, with *p* < 0.05 being considered statistically significant. To observe whether age, number of drugs, days of treatment and sex of patients were associated with the prevalence of inappropriate prescription based on STOPP and START criteria, logistic regression was employed. Thus, by quantifying the category of variables, STOPP/START criteria were considered dependent variables, and the remaining variables were considered independent variables.

### 2.5. Ethical Considerations

This study has been conducted in accordance with the Helsinki Declaration and its later amendments and has been approved by the Ethics Committee of “Victor Babes” University of Medicine and Pharmacy (no. 7/2016).

## 3. Results

With an average age of 74.1 ± 6.95 years, 43% of the total prescriptions included (n = 1498) were prescribed for male and 57% for female patients. Table 1 shows the demographic distribution of EMPCCs: a higher share of prescriptions was collected from the urban area of TM County (≅78%, n = 1170), compared with that of AR County (≅22%, n = 328).

The prescriptions’ characteristics (prescribing physician, number of prescribed drugs, and days of treatment) are presented in Table 2. The majority of EMPCCs (88.65%) were prescribed by the general practitioner, with a predominant overall prescription duration of 30 days of treatment (in 78.3% of cases) and an average number of drugs of 4.7 ± 1.51 per prescription. Notably, a significantly increased proportion of EMPCCs were prescribed by specialist physicians in the TM urban area, compared with the AR urban area (97.6% versus 2.4%, *p* < 0.001). Also, the average number of prescribed drugs was higher in TM County than in AR County (4.8 ± 1.52 versus 4.3 ± 1.40, *p* < 0.001).

Table 3 shows the frequency of diagnostic codes in the collected EMPCCs, as per the national diagnostic classification system. Cardiovascular (CV) and cerebrovascular diseases were identified as the most common pathologies, in particular, essential arterial hypertension (79.7%), ischaemic coronary heart disease (43.8%) and other cerebrovascular diseases (16%). Dyslipidaemia was also highly prevalent, with a percentage of 52.3% of all EMPCCs in both counties. Lastly, 13.5% of diagnoses were represented by gastrointestinal tract disorders, such as gastritis and duodenitis, and 9.2% by benign prostatic hyperplasia.

Out of the 26 STOPP criteria, 17 of them were identified in our EMPCCs (Table 4). A total of 23.6% of the included EMPCCs have been identified as having an overly prolonged treatment period, with the percentage being higher in AR County (26.7%). The second most prevalent STOPP criterion was the use of neuroleptics (14.6%), with TM County facing this problem in 16.5% of cases. Zopiclone prescription was identified in 14% of the EMPCCs, with a considerable difference between the two counties (15.7% for TM County versus 6.7% for AR County). A percentage of 11.5% of EMPCCs were identified as containing active components with no evidence-based clinical indication. This STOPP criterion was significantly more common in AR County (33.3%) compared to TM County (6.3%), with a *p* value < 0.001. In total, 8.9% of the medical prescriptions had a duplicate drug pharmacological class, with a greater instance of duplication in TM County, compared with AR County (9.4% vs. 6.7%). The prescription of COX2-selective non-steroidal anti-inflammatory drugs (NSAIDs) in patients who have cardiovascular disease (CVD) accounted for 6.4% of cases, with minor differences between the two counties. Regarding the prescriptions having theophylline recommended as monotherapy in chronic obstructive pulmonary disease (COPD), a percentage of 5.1% of EMPCCs were identified, with 4.7% in TM County and 6.7% in AR County. Regarding the treatment for cardiovascular disorders, associations of angiotensin-converting enzyme (ACE) inhibitors and angiotensin receptor blockers (ARBs) in patients with hyperkalaemia, and the prescription of loop diuretics as the first-line treatment for hypertension, were the most frequently encountered STOPP criteria in TM County (5.5% and 3.1%, respectively). In AR County, these STOPP criteria were not identified among the EMPCCs (0%). Another STOPP criterion absent in AR County (0%) was the administration of neuroleptics with anticholinergic effects, while in TM County, the criterion was found in 6.7% of cases (*p* = 0.042).

Table 4 presents the 17 STOPP criteria identified by analysing the EMPCCs. The main criteria are the indication of medicines, cardiovascular system, central nervous system and psychotropic drugs, respiratory system, and gastrointestinal system. The STOPP criteria with the highest prevalence was prescribed beyond the recommended treatment duration. The prescribing tendencies of duplicate drug classes or drugs and of medications with no evidence-based clinical indication were also high in percentage. A high prevalence in prescribing nervous system medicine was also found, more specifically of neuroleptics, which may increase the risk of falling, and for zopiclone, with a risk of overdosing due to the multiple doses prescribed.

Table 5 shows that 18.58% of the EMPCCs in the two counties contained PIMs (*p* = 0.099), with a slightly higher prevalence of PIMs in TM County (18.63%) than in AR County (18.40%).

Regarding START prevalence, Table 6 presents the analysis of PPOs. Hence, PPOs were found in 49.20% of EMPCCs (*p* = 0.037), with TM County having a lower prevalence of them (47.00%) compared with AR County (57.05%).

In terms of the START criteria, 12 criteria were detected within our EMPCCs (Table 7). Strikingly, 47.4% of prescriptions lacked statin treatment, despite the patient’s history of coronary, cerebral, or peripheral vascular disease; notably, AR County (57.9%) has a significantly higher prevalence (*p* = 0.007) of this criterion, compared with TM County (41.3%). In total, 24.5% of the EMPCCs did not have a recommendation for beta-blockers in patients with ischemic heart disease, with a greater share in AR County (28.1%) than in TM County (22.4%). Significant differences between the two counties were recorded in terms of antiresorptive or bone anabolic medication (15.8% in TM County versus 0% in AR County, *p* < 0.001), with TM County lacking effective treatment for osteoporosis. Regarding the recommendation of antihypertensive drugs when blood pressure > 160/90 mmHg (6.1%), a greater percentage was recorded in TM County (7.7%) than in AR County (3.5%). Another START criterion identified in 4.5% of EMPCCs was the omission of an inhaled beta2-agonist or antimuscarinic bronchodilator in COPD or mild-to-moderate asthma, with TM County having more omissions (6.1%) than AR County (1.8%). Prescription omission of a bisphosphonate in association with vitamin D and calcium in patients with long-term systemic corticosteroid therapy was observed in 1.6% of cases, with a higher prevalence in AR County (3.5%) than in TM County (0.5%).

The prevalence of STOPP criteria is significantly associated with the following: female gender, prescriptions provided by specialists, and the increased number of drugs prescribed (Table 8).

Table 9 shows that the prevalence of START criteria is also significantly associated with the female gender, as well as with the prescriptions provided by general physicians, a lower number of drugs prescribed, and a shorter treatment period.

In terms of observed prescription errors (Table 10), TM County (3.2%) has a slightly higher prevalence of overdosed medication (3.1%) (Table 11) than AR County (2.4%). Regarding the EMPCCs which contain underdosed medication (2.3%) (Table 12), TM County has a lower share (2.2%) compared to AR County (2.4%). A significant difference was observed in the prevalence of wrong diagnostic codes (2.8%, *p* < 0.001)(Table 13), with AR County having a greater rate (6.7%) than TM County (1.2%).

Table 14, Table 15 and Table 16 present examples of STOPP criteria found in the analysed prescriptions.

## 4. Discussion

The latest report on the state of health in Romania reveals extremely high rates of preventable and treatable mortality, as opposed to the EU’s average. The main causes of mortality include ischaemic heart disease, pneumonia, stroke, and cancer. Given the ageing population [5,34] and the declining natality rate (crude birth rate—defined as the number of births per 1.000 people—has dramatically decreased from 25.2 births in 1950 to 10.1 births in 2021) [35], there is an increasing need to monitor and optimise the medication of elderlies. This is essential to improve their life expectancy, reduce hospitalisation and mortality rates, and ease the burden on healthcare services. According to the same report, Romania’s health spending in 2021 was less than half of the EU average (EUR 1663 per capita, versus EUR 4029 per capita), with a significant part of the funds being allocated to inpatient care at the expense of outpatient care. Moreover, the Romanian healthcare system is focused on hospital services, with insufficient use of primary care [5]. Yet, there is strong evidence supporting the importance of primary care: it determines a fair distribution of healthcare in population subgroups, contributes to the prevention of health disorders and mortality, and may even lower the costs of health services [36].

Given that the issues of drug overuse and misuse have been addressed in our previous work [27,28], herein we will exclusively discuss the matter of underuse, with an emphasis on the most commonly utilised classes of drugs:

1. Statins;
2. Beta-blockers;
3. Antiresorptive medications;
4. Beta2-agonists;
5. Psychotropic drugs.

### 4.1. Statins

In our study, the most prevalent PPO was the lack of statin treatment in patients with a history of coronary, cerebral, or peripheral vascular disease. HMG-CoA reductase inhibitors, often referred to as statins, are one of the most important therapies for the prevention and treatment of atherosclerotic CVDs [37]. Their hypolipidemic and pleiotropic properties make them ideal candidates for use in primary and secondary CV prevention [38]. Hypolipidemic activity results in lowering the plasma concentrations of low-density lipoprotein cholesterol (LDL-C), total cholesterol, and triglycerides and increasing the concentration of high-density lipoprotein cholesterol (HDL-C). In terms of pleiotropic effects, statins play a role in stabilising atheroma plaque, reducing inflammation (by decreasing the levels of pro-inflammatory cytokines and C-reactive protein [39]), improving endothelial function and preventing thrombogenicity (by decreasing platelet activity and reducing the synthesis of thromboxane A2 [39]). Another important pleiotropic effect is the antioxidant action [40], which involves (1) the inhibition of the activity of NADPH—oxidase and myeloperoxidase [41]; (2) the upregulation of the antioxidant enzyme system represented by glutathione peroxidase, superoxide dismutase, and catalase [42]; and (3) the modulation of nuclear factor erythroid 2-related factor 2 (Nrf2)/HO-1 signalling pathway with major implications in the cellular response to oxidative stress [43].

Although guidelines recommend statins in primary and secondary prevention of CVD, clinical practice, including our study, reveals poor identification of patients with CV risk and, consequently, suboptimal administration of statins [44,45]. A study conducted between 2020 and 2021 showed that in Central and Western Europe—particularly in primary care—the implementation of guidelines is low and LDL-C control in patients at high or very high CV risk is suboptimal [46]. Bradley C. et al. also brought attention to the underuse of statins: 27% of study participants (n = 1511) did not follow a statin treatment, despite being eligible for treatment [47]. A 2021 prospective study based on START/STOPP criteria v2 identified 58.1% PPOs among hospitalised patients in an academic hospital (n = 16,687), with statin therapy being in the top five in terms of prevalence [48]. Mohammed et al. showed that omission of statin prescriptions was common at both hospital admission (13% of all PPOs, n = 846) and discharge (18% of all PPOs, n = 846), without a justifiable explanation [49]. Numerous factors might lead to underutilisation: physician’s lack of knowledge of lipid guidelines and the proven long-term benefits of the statins, poor patient–physician relationship, patient’s advanced age, lack of adherence, concern about side effects, multiple drug interactions of statins, and costs [50].

There are also differences in adherence to primary and secondary prevention. In the case of primary prevention, most adherence issues arise from patients’ inability to perceive direct symptomatic results from statins, making them less likely to recognise their benefits [51]. Other reasons for non-adherence include patients’ misunderstanding of the need to follow the treatment; treatment costs; patients’ distrust or suspicion regarding the real reasons behind the doctors’ prescriptions; and the high prevalence of depression among patients, especially post-COVID-19 [52,53,54]. In this context, secondary prevention requires a higher rate of statin use in patients with a history of CV events [55]. Adherence and prescription practices also appear to vary by sex. Clinicians prescribe statins more to men than to women, as men are more likely to adhere to the recommendation and continue medications [56]. A 2019 study by Musich S. et al. in 2019 found discrepancies between the sexes: 18% of men with CVD did not receive a statin recommendation, and 26% were not adherent to treatment. Compared to male patients who take high-intensity statins, those who do not take statins have a mortality rate of 65%. Conversely, 29% of females with CVD do not take statins, 27% are non-adherent, and the mortality rate is 41% lower in women taking high-intensity statins [57]. Under-prescription is particularly common among the elderly, due to the large number of drugs in their therapeutic plan and the prevalence of frailty [58]. The suboptimal prescription of statins might also stem from concerns about the potential onset or worsening of cognitive functions and the development of Alzheimer’s disease, particularly in elderly people. However, statins’ negative effects on cognitive function are questionable and have not been confirmed by conclusive data [59,60]. Low adherence to statin treatment and suboptimal use also relates to the occurrence of side effects, which occur regardless of age and gender, liver damage [47], muscle pain, and, in some cases, new-onset diabetes mellitus [61,62,63].

Given that most statins are metabolised via cytochrome P450 microsomal enzymes, namely, CYP3A4, CYP3A5, CYP2C9 and CYP2C19, numerous interactions with other enzyme inhibitors or inducers may occur. Interactions are significantly more common in patients under polymedication, especially with fenofibrate, amiodarone, acenocumarol, calcium channel blockers, spironolactone, digoxin, valsartan, and budesonide [64]. The polymorphism of some genes, such as mutations of genes encoding the influx/efflux transporters in hepatocytes, can affect the pharmacokinetics of statins. Other genetic modifications alter the pharmacodynamics of statins, leading to reduced therapeutic efficacy or increased risk of adverse effects. The genotype of LDL-C receptors can also influence the response to statin treatment [65]. Another problem regarding suboptimal statin use and under-prescription is access to medical care and healthcare coverage, especially in disadvantaged areas [66].

### 4.2. Beta-Blockers

Another START version 2 criteria is prescribing beta-blockers to patients with ischemic heart disease [22]. In our study group, this START criterion was the second most frequently encountered PPO. Our results align with Primejdie et al., who also found that beta-blockers are underused in both ambulatory and institutionalised elderly people’s pharmacotherapy [67]. Due to their multiple benefits, including lowering the 5-year all-mortality rates, beta-blockers should be prescribed more [68]. Beta-blockers are classified in three generations: (1) first-generation beta-blockers (e.g., propranolol)—non-selective and act by blocking β1 and β2 receptors; (2) second-generation beta-blockers (e.g., metoprolol)—cardio-selective and act by blocking β1 receptors; and (3) third-generation beta-blockers (e.g., nebivolol)—cardio-selective and have additional vasodilatory effects [69]. Inhibiting β1-receptors in the heart [68,70] minimises the unwanted effects of adrenergic stimulation which are associated with negative outcomes such as arrhythmias, cardiac remodelling, myocardial fibrosis, systolic dysfunction, and heart failure [68,70]. The additional vasodilating and antioxidative properties of third-generation beta-blockers are independent of β1-receptor blockade and have been shown to indirectly improve endothelial dysfunction. At the endothelial level, nebivolol enhances the activity of eNOS, stimulates NO release, and decreases vascular tonus. Moreover, nebivolol was shown to improve endothelial function by reducing vascular oxidative stress through NAD(P)H oxidase and ROS inhibition [69,71,72,73].

Beta-blockers are indicated in patients with stable coronary artery disease, exercise-induced angina, ACS without hypotension, or decompensated heart failure, and they are essential in managing patients with heart failure with reduced ejection fraction [68,70]. Beta-blockers appear to have similar efficiency in angina control compared to other classes of antianginal therapy, such as calcium channel blockers. However, the administration of beta-blockers as first-line antianginals is associated with a lower ratio of 5-year all-cause mortality in patients suffering from stable coronary artery disease who had ACS in the previous year. Conversely, calcium channel blockers showed no mortality benefit [68]. Moreover, the POISE-1 randomised controlled trial showed that perioperative administration of beta-blockers (metoprolol) reduced the risk of atrial fibrillation and ACS [74]. Lindgren et al. discovered that the therapy with cardio-selective beta-blockers was associated with long-term benefits and a lower risk of myocardial infarction and major adverse cardiovascular events after coronary artery bypass grafting [75].

Despite their beneficial effects, beta-blockers can have a negative impact on patients’ lives, psychological health, and relationships, leading to low medication adherence. The risk of adverse events is higher in patients receiving high-dose regimens, patients under chronic treatment, and the elderly population. The main cause of beta-blocker noncompliance is sexual dysfunction, manifested as erectile dysfunction in men and loss of libido in women; this adverse effect is dose-dependent and more frequent in regimens containing first-generation beta-blockers. Given that penile erection is a neurovascular manifestation that depends on neuronal and vascular NO release, it is recommended to substitute first-generation beta-blockers with third-generation beta-blockers. The latter have vasodilatory effects on genital tissue which translate into a lower risk (carvedilol) or null risk (nebivolol) of sexual dysfunction [76,77]. Furthermore, treatment with beta-blockers may increase the risk of depression and adverse neuropsychiatric events, such as fatigue, sleep disturbances, night terrors, visual hallucinations, delirium, psychosis, Parkinson’s disease, and an increased risk of falls [78,79].

Inter-individual variability of the response to beta-blocker pharmacotherapy can be the result of (1) mutations in CYP2D6 encoding genes—which affect the metabolism of beta-blockers (e.g., metoprolol—CYP2D6 enzyme is responsible for 70–80% of its biotransformation to inactive metabolites; carvedilol and nebivolol—beta-blockers marketed as racemic mixtures, and their enantiomers have distinct activities and are metabolised through different pathways; labetalol; and timolol) [80,81]; and (2) polymorphism of the genes encoding β1- and β2-adrenergic receptors—which can affect treatment response [82].

Given Romania’s high mortality rate due to ischemic heart disease (18.8%) (5), clinicians should promote appropriate prescribing of beta-blockers in order to minimise underuse and decrease morbidity and mortality due to CVD. To do so and in order to reduce the gap of knowledge between scientists and practitioners, clinicians should participate in continuing medical education. To ensure optimal prescribing practices, special attention should be devoted to the pharmacological properties of beta-blockers: (1) hydrophilic beta-blockers can be recommended to elderly patients; (2) lipophilic beta-blockers can be useful in managing neuropsychiatric disorders such as migraines, tremors, anxiety, and obsessive–compulsive disorder; (3) nebivolol is the best option in treating patients with erectile dysfunction; and (4) CYP2D6 genotyping predicts CYP2D6 metabolism and could guide optimal prescription of beta-blockers, and in case of mutations of CYP2D6 encoding genes, acebutolol, atenolol, betaxolol, bisoprolol, or nadolol could be an alternative because these molecules are not metabolised by the CYP2D6 isoenzyme [80].

We found a high prevalence of potential prescribing omissions (PPOs) in AR versus TM (57.05% vs. 47.00%, *p* = 0.037), with statins and beta-blockers being the leading underused medicines. Several facts could explain differences in prescribing practices between the two counties: a. more developed counties with bigger cities (TM County population: 650,533 vs. AR County: 410,143) usually have better (healthcare) infrastructure and life expectancies [1,2,83,84]; b. moreover, as already stated, TM County has almost five times the number of ambulatory clinicians compared with AR which may have an impact on the public healthcare access and services provided [29]; and c. TM County is one of the main university centres in Romania, which may lead to better education (including physicians updated on the latest guidelines and trends regarding prescribing practices), the possibility of more job opportunities, and higher income. These aspects may contribute to higher access to qualitative medical services, drug availability, affordability [85], and, thus, a better and safer quality of life.

### 4.3. Antiresorptive Medications

We have also found antiresorptive therapy to be under-prescribed; therefore, we will focus on the reasons why preventive and curative antiosteoporotic regimens should be better addressed in the Romanian elderly population. In recent years, the prevalence of osteoporosis has increased, as has the disease’s economic burden. [86,87,88]. It was reported that 18.3% of the world’s population is affected by osteoporosis, with women having a higher risk of developing the disease than men [89]. In Europe, it is estimated that 5.3% of people have osteoporosis, and the majority of European countries have experienced a rise in the number of patients with the condition [87]. In Romania, the prevalence of osteoporosis is slightly lower than the mean European value, although many patients may be underdiagnosed—an aspect that our study also highlights. A SCOPE report states that 20.5% of Romanian women and 6.2% of men aged 50 years or more are diagnosed with osteoporosis [90].

In Romania, the number of high-fracture-risk individuals that do not receive antiosteoporosis therapy is high compared to other European countries. Between 2019 and 2034, a 15% rise in the number of fragility fractures was expected, which will affect the healthcare budget [90]. Romania ranked at the bottom out of 29 EU countries in terms of osteoporotic fracture costs per patient [87,90].

Several guidelines bring updated guidance on the prevention, diagnosis, evaluation, and treatment of osteoporosis [91,92,93,94]. Bisphosphonates, a class of antiresorptive drugs, are efficient in reducing the risk of vertebral, nonvertebral, hip, and wrist fractures. Within the pharmacological class, zoledronate has the highest efficacy in reducing vertebral, hip, and wrist fractures, while risedronate is the most effective in reducing nonvertebral fractures [95,96]. Another potent antiosteoporosis agent is denosumab, a human monoclonal antibody targeting RANKL, the main mediator of bone resorption and a major player in osteoclast development, activity, and apoptosis [92,93]. Denosumab can be used to treat osteoporosis in patients with a high fracture risk or in those intolerant to or who have failed other antiosteoporosis therapies. Oral bisphosphonates (e.g., alendronate, zoledronic acid, and risedronate, but not ibandronate) should be the initial treatment in high-risk patients, with denosumab being an alternative therapy to reduce fracture risk. Teriparatide, abaloparatide for up to 2 years, and romosozumab for up to 1 year should be the first-line therapy for very-high-risk patients [93,94].

Calcium and vitamin D have an essential role in bone homeostasis. Moreover, vitamin D is highly important for calcium homeostasis, facilitating its absorption [93,97]. Unfortunately, in Europe, the elderly have an insufficient intake of calcium and vitamin D, predisposing them to osteoporosis [88,97]. In addition, an optimal dietary protein intake is also essential, considering that proteins represent approximately one-third of bone mass and that elderly patients are predisposed to sarcopenia [91,95]. Magnesium and zinc are also essential nutrients for bone health [88,97].

### 4.4. Beta2-Agonists/Anticholinergic Medicines

One other START criterion we have identified in our EMPCCs is the underuse of inhaled beta2-agonists or antimuscarinic bronchodilators in COPD or mild-to-moderate asthma. Beta2-agonists and muscarinic antagonists are two of the main classes of bronchodilators [98]. Beta2-agonists exert their bronchodilator effects by binding to β2-adrenoreceptors in the smooth muscle cells lining the airways [99]. They are routinely used in asthma and in COPD [100]. Inhaled long-acting beta2-agonists (LABAs) and short-acting beta2-agonists (SABAs) are used as maintenance treatment and in exacerbations, respectively [99]. The ADRB2 gene polymorphism seems to impact the safety profile of beta2-agonists in asthmatic patients [101]. Over-prescription of SABAs in the elderly is associated with poor asthma outcomes, triggering adverse events such as airway hyper-responsiveness, frequent exacerbations, and increased mortality [102,103]. Contrarily, monotherapy with beta2-agonists in COPD appears to be risk-free and to have a positive impact on the evolution of the disease [104]. On the other hand, antimuscarinic drugs, also known as muscarinic antagonists, act by blocking the three muscarinic receptors, particularly M3 receptors [105]. Long-acting muscarinic antagonists (LAMAs) were shown to improve symptoms and health status in obstructive lung diseases by reducing the exacerbation rate [106,107].

There are three processes contributing to the pathophysiology of asthma: mucus hyperproduction, airway remodelling, and bronchial hyperresponsiveness [108]. Given the disease’s inflammatory characteristic, inhaled corticosteroids (ICSs) are regarded as the main therapeutic measure in asthma [109]. The association of ICSs with LABAs, LAMAs, or leukotriene receptor antagonists was shown to lower ICS dosages, control the symptoms, and reduce the risk for exacerbations [110]. The Global Initiative for Asthma (GINA) systematically presents the most recent international recommendations for asthma treatment. In their Global Strategy for Asthma Management and Prevention report, GINA proposes a five-step approach, starting with low doses of ICS + LABA and adding LAMA in step 5 [111]. Data from the SABA Use IN Asthma (SABINA) global programme show that 40% of asthma patients overuse SABA relief inhalers, leading to poor clinical outcomes [112].

In COPD, the persistent inflammation and destruction of pulmonary tissues lead to disruptions in the alveolar homeostasis [113]. Ever since their introduction, inhaled long-acting bronchodilators such as LAMAs and LABAs have remained the gold standard for therapy in the management of symptomatic COPD [114]. Of note, the association of the two bronchodilator classes has shown substantial benefits, with increased efficacy and safety [115]. In 2023, the Global Initiative for Chronic Obstructive Lung Disease (GOLD) released the fifth major revision of its comprehensive report, which considered the most recent publications on the diagnosis, management, and prevention of COPD. Here, monotherapy with inhaled long-acting bronchodilators such as LABAs or LAMAs is presented as the first intention strategy in COPD with infrequent annual exacerbations. If the response to the initial treatment deteriorates over time, GOLD recommends the dual bronchodilator therapy LABA + LAMA, with an emphasis on a single inhaler regimen. The triple therapy LABA + LAMA + ICS is initiated only in specific situations, for patients associating asthma or high blood eosinophil count (≥300 cells/μL). Notably, when ICSs are pressingly needed, GOLD advises against the use of LABA + ICS, and instead recommends the triple therapy, as it was proven to be superior in terms of effectiveness [116]. However, despite current guidelines, bronchodilator therapy is often associated with an ICS (either LABA + ICS or LABA + LAMA + ICS) in the absence of a clear indication [117].

### 4.5. Psychotropic Drugs

Despite the fact that we did not find too many START criteria concerning psychotropic drugs, we consider that mental health problems are currently insufficiently addressed and debated in Romanian society [118]. Mental health is an integral component of health and can be defined as a mental state of well-being that enables people to cope with stressful conditions. WHO’s report on the world’s mental health stated that one in eight people is suffering from a mental health condition, making these disorders extremely common worldwide. Mental health issues are not properly addressed, affecting the life expectancy, quality of life, and productivity of the people struggling with these conditions [119]. Overall, Romania reports the lowest percentage of mental disorders among European countries, with anxiety and depression being the most frequent. However, the depression risk ratio reported by Romanians is 5% higher than the European average [5]. It is well known that anxiety and depression are particularly common among elderly people and can exacerbate insomnia [120]. Therefore, the use of antidepressants such as trazodone in the elderly population is considered beneficial and should be encouraged, as they exhibit a lower risk of dependence than other sedatives, including benzodiazepines and “Z” drugs [120].

Mental disorders are presumably underdiagnosed and undertreated [119] and patients are hesitant to receive specialised care due to a lack of awareness, stigma, and negative preconceptions that are pervasive in society, the workplace, the media, and even in healthcare settings [5,118,119]. Stigma affects patients’ adherence to treatment and leads to higher comorbidity and mortality rates [118]. Investing in mental health, facilitating patient access to treatment, developing guidelines to address stigma, and adopting policies to prevent mental disorders are all needed in Romanian elderly patients [118,119].

### 4.6. Gender Differences in Prescribing Patterns in Elderly

Our research also underscores the urgent need to address the significant gender disparities in prescribing patterns for the elderly. We found that both PIMs, as well as PPOs, are significantly associated with gender, more specifically with female patients. Our findings are consistent with the scientific literature, which stipulates that women are more prone to use healthcare services and polypharmacy, and to self-medicate, as several articles describe these healthcare gender stereotypes [121,122,123]. Physiological gender differences, which lead to variations in body composition and drug metabolism, could also contribute to inappropriate medication use in women and, thus, to a high prevalence of side effects [124]. Moreover, several publications highlighted that older women may receive different care levels than men, thus having different outcomes [125]. In line with this statement is a study published by Kwak M. and collaborators that addresses the differences in informal care in community-dwelling older adults, highlighting the fact that in the United States and China, older women receive better informal care than men, compared with those from South Korea (although this can vary among countries) [126].

### 4.7. Future Directions and Limitations

Sipos et al. appraised potentially inappropriate prescribing (PIP) practices among discharged elderly people. They found that the major risk factors for PIP are polypharmacy—a prescription of over 10 medications—and CVDs such as hypertension and heart failure [127]. While elderly people usually take 5 or more medications, institutionalised patients are prescribed an average of 7–8 medications [128,129]. Pharmacotherapy optimisation should involve a patient-centred approach [15,130], particularly for patients over 65 years old with comorbidities and complex treatment regimens. In Romania, as in other Eastern European countries, pharmaceutical services are mostly traditional and focused on medication dispensing, patient counselling, and compounding, while medication review services are nearly absent. Yet, the implementation of this service is of great importance because it could improve medication adherence and reduce medication-related issues [131]. In brief, our findings are consistent with the literature: PPOs have a higher prevalence than PIMs in elderly people [130]. Thus, to prevent negative health outcomes, the matter of under-prescribing should be treated at least as seriously as over-prescribing [132,133]. Moreover, in future research papers, we plan to address and explore the possible underlying causes that could explain the prescribing differences (PIMs and PPOs) between the two countries.

This study has several limitations. Firstly, we were unable to apply all the STOPP/START criteria in the absence of clinical records, laboratory data, and information regarding the use of OTCs and supplements by patients. Secondly, given the retrospective nature of our study, we also lack information regarding the long-term effects (hospitalisation rates and other healthcare-related costs) of the identified PIMs. Lastly, the outcomes of our study are limited to the urban areas of Western Romania, as the data for rural areas have been previously published [28]. The present findings should be interpreted with caution due to the limitations of the data set and are applicable only to the areas where the study was conducted. Thus, the results of the present study cannot be generalised for the entire country.

## 5. Conclusions

Underuse of statins, beta-blockers, antiresorptive therapy, beta2-agonists and anticholinergic bronchodilators were among the most identified drug-related problems in the Romanian elderly population. In order to reduce iatrogenic risks in an ageing population, Romania urgently needs to reinforce primary care and provide physicians with periodic training to ensure they are updated on the latest guidelines. In this regard, the implementation of electronic patient records would be tremendously beneficial for securing the medical act and enabling patient-centred pharmacotherapy. Lastly, to address the issue at its core, the implementation of medication review services performed by clinical pharmacists should be encouraged by the healthcare system and supported through reimbursement by the national assurance company.

## Figures and Tables

**Table 1:** Prescriptions’ demographic data.

General Information on the Population Studied	Gender		Age (Years)
	Female	Male	
Total (%)	57.0%	43.0%	74.1 ± 6.95
AR urban (%)	22.0%	21.7%	73.2 ± 6.65
TM urban (%)	78.0%	78.3%	74.3 ± 7.02
p ^sig^	0.928 ^a,is^		0.084 ^b,is^

Notes: AR—Arad County; TM—Timis County; ^a^—chi^2^ tests; ^b^—Mann–Whitney U test; ^is^—insignificant difference; ^sig^—significant difference.

**Table 2:** Prescriptions’ characteristics.

General Information on the Prescriptions	Physician		Number of Prescribed Drugs (as Mean)	Days of Treatment		
	Generalist	Specialist		30	60	90
Total (%)	88.65%	11.35%	4.7 ± 1.51	78.3%	1.3%	20.4%
AR urban (%)	24.4%	2.4%	4.3 ± 1.40	25.9%	100.0%	1.3%
TM urban (%)	75.6%	97.6%	4.8 ± 1.52	74.1%	0.0%	98.7%
p ^sig^	<0.001 ^a,s^		0.001 ^b,s^	<0.001 ^a,s^		

Notes: AR—Arad County; TM—Timis County; ^a^—chi^2^ tests; ^b^—Mann–Whitney U test; ^s^—significant difference; ^sig^—significant difference.

**Table 3:** The diagnostic code description within the classification system encountered in the prescriptions and its corresponding percentage.

Diagnostic (Code—Description)	Total (%)
Cardiovascular and cerebrovascular disorders	
**453—Essential hypertension**	**79.7**
458—Angina pectoris	0.9
**462—Chronic ischemic heart disease**	**43.8**
476—Cardiomyopathy	4.0
480—Unspecified stroke	1.3
**481—Other cerebrovascular diseases**	**16.0**
485—Other peripheral vascular diseases	4.0
491—Varicose veins of lower limbs	6.9
Nervous system disorders	
300—Dementia without specification (presenile, senile)	3.2
303—Other mental disorders due to brain injury	1.1
312—Schizophrenia	0.0
321—Depressive episode	4.4
325—Anxiety disorders	3.7
350—Hyperkinetic disorders	4.0
364—Parkinson’s disease	2.7
368—Alzheimer’s disease	2.0
373—Epilepsy	0.9
Metabolic and endocrine disorders	
203—Iron deficiency anaemia	0.3
236—Hypothyroidism due to subclinical iodine deficiency	1.1
**289—Metabolism disorders of lipoproteins and other dyslipidaemia**	**52.3**
290—Metabolism disorders of purine and pyrimidine	5.6
630—Gout	1.3
Respiratory system disorders	
526—Other chronic obstructive pulmonary diseases	3.5
527—Asthma	2.8
Gastrointestinal tract disorders	
**559—Gastritis and duodenitis**	**13.5**
Musculoskeletal system disorders	
632—Polyarthritis	5.5
648—Spondylosis	0.3
661—Osteoporosis	6.4
Genitourinary system disorders	
**701—Benign prostatic hyperplasia**	**9.2**

Notes: Bold highlights the most encountered diagnostic codes.

**Table 4:** STOPP criteria identified in elderly patient prescriptions.

Summarised STOPP Criteria	Total (%)	Arad (%)	Timis (%)	p ^sig^ (Chi^2^)
1. Treatment prescribed beyond recommended duration	23.6	26.7	22.8	0.831 ^is^
2. Neuroleptics—with increased risk of falls	14.6	6.7	16.5	0.281 ^is^
3. Zopiclone—overdosed, multiple doses administered	14.0	6.7	15.7	0.324 ^is^
4. No evidence-based clinical indication	11.5	33.3	6.3	<0.001 ^s^
5. Duplicate drug class/drug prescriptions	8.9	6.7	9.4	0.912 ^is^
6. COX2-selective NSAIDs in patients with cardiovascular disease	6.4	6.7	6.3	0.738 ^is^
7. Theophylline as monotherapy in COPD	5.1	6.7	4.7	0.989 ^is^
8. ACE inhibitors/ARBs in patients with hyperkalaemia	4.5	0.0	5.5	0.411 ^is^
9. Loop diuretics—as first-line treatment for hypertension	2.5	0.0	3.1	0.743 ^is^
10. Aldosterone antagonists associated with drugs that spare potassium	1.9	0.0	2.4	0.903 ^is^
11. Loop diuretics for arterial hypertension in patients with urinary incontinence	1.9	0.0	2.4	0.903 ^is^
12. Neuroleptics with anticholinergic effects	1.3	6.7	0.0	0.042 ^s^
13. PPIs in peptic ulcer disease/erosive peptic esophagitis for more than 2 months at maximum therapeutic dosage	1.3	0.0	1.6	0.841 ^is^
14. Antithrombotic combinations of drugs—risk of bleeding	0.6	0.0	0.8	0.439 ^is^
15. Neuroleptics administered as hypnotics	0.6	0.0	0.8	0.439 ^is^
16. NSAIDs or colchicine used in gout for more than 3 months	0.6	0.0	0.8	0.439 ^is^
17. NSAIDs with corticosteroids without prophylactic PPI	0.6	0.0	0.8	0.439 ^is^

Abbreviations: ACE—angiotensin-converting enzyme; ARBs—angiotensin receptor blockers; PPI—proton pump inhibitor; NSAID—non-steroidal anti-inflammatory drug. Notes: ^is^—insignificant difference; ^s^—significant difference; ^sig^—significant difference.

**Table 5:** STOPP prevalence (PIM—potentially inappropriate medication).

Total PIM	AR Urban	TM Urban	p ^sig^ Using Chi^2^ Test
18.58%	18.40%	18.63%	0.099 ^is^

Notes: ^is^—insignificant difference; ^sig^—significant difference.

**Table 6:** START prevalence (PPOs—potential prescribing omissions).

Total PPO	AR Urban	TM Urban	p ^sig^ Using Chi^2^ Test
49.20%	57.05%	47.00%	0.037 ^s^

Notes: ^s^—significant difference; ^sig^—significant difference.

**Table 7:** START criteria identified in elderly patient’s prescriptions.

Summarised START Criteria	Total (%)	AR (%)	TM (%)	P (Chi^2^)
**1. Statins with history of coronary, cerebral, or peripheral vascular disorders**	**47.4%**	**57.9%**	**41.3%**	**0.007 ^s^**
**2. Beta-blockers administered in ischaemic heart disease**	**24.5%**	**28.1%**	**22.4%**	**0.323 ^is^**
**3. Bone antiresorptive or anabolic therapy in documented osteoporosis**	**10.0%**	**0.0%**	**15.8%**	**<0.001 ^s^**
**4. Antihypertensive drugs when blood pressure > 160/90 mmHg**	6.1%	3.5%	7.7%	0.216 ^is^
**5. Beta2-agonist inhaled or antimuscarinic bronchodilator for COPD or mild-to-moderate asthma**	**4.5%**	**1.8%**	**6.1%**	**0.140 ^is^**
**6. Bisphosphonates/vitamin D/calcium in long-term systemic corticosteroid therapy**	1.6%	3.5%	0.5%	0.119 ^is^
7. Vitamin D and calcium in osteoporosis or history of fractures	1.6%	3.5%	0.5%	0.119 ^is^
8. SSRI/SNRI or pregabalin with severe persistent anxiety	1.0%	0.0%	1.5%	0.480 ^is^
9. PPI with severe gastro-oesophageal reflux disease	0.6%	1.8%	0.0%	0.247 ^is^
10. ACE inhibitors with history of coronary artery disease and systolic heart failure	0.3%	0.0%	0.5%	0.770 ^is^
11. Appropriate beta-blocker in systolic heart failure	0.3%	0.0%	0.5%	0.770 ^is^
12. Alpha-1 receptor blocker with symptomatic prostatism	0.3%	0.0%	0.5%	0.770 ^is^

Abbreviations: ACE—angiotensin-converting enzyme; COPD—chronic obstructive pulmonary disease; SSRIs—selective serotonin reuptake inhibitors; SNRIs—serotonin and norepinephrine reuptake inhibitors; PPI—proton pump inhibitor. Notes: ^is^—insignificant difference; ^s^—significant difference. Bold highlights the most encountered diagnostic codes.

**Table 8:** Variables associated with STOPP prevalence.

Variables in the Equation		Physician	Gender	Age	No. of Drugs	Days of Treatment	Constant
**B**		−1.318	0.47	0.008	0.195	−0.003	−2.072
**S.E.**		0.279	0.205	0.014	0.07	0.004	1.072
**Wald**		22.344	5.285	0.306	7.86	0.48	3.738
**Df**		1	1	1	1	1	1
**Sig.**		0	0.022	0.58	0.005	0.488	0.053
**Exp(B)**		0.268	1.601	1.008	1.215	0.997	0.126
95% C.I.for EXP(B)	Lower	0.155	1.072	0.98	1.06	0.988	
	Upper	0.462	2.391	1.036	1.393	1.006	

**Table 9:** Variables associated with START prevalence.

Variables in the Equation		Physician	Gender	Age	No. of Drugs	Days of Treatment	Constant
**B**		3.235	0.372	0.022	−0.268	−0.012	−3.908
**S.E.**		0.605	0.17	0.012	0.058	0.004	1.073
**Wald**		28.618	4.78	3.295	21.046	10.8	13.257
**Df**		1	1	1	1	1	1
**Sig.**		0	0.029	0.069	0	0.001	0
**Exp(B)**		25.405	1.451	1.022	0.765	0.988	0.02
95% C.I.for EXP(B)	Lower	7.766	1.039	0.998	0.682	0.981	
	Upper	83.11	2.026	1.046	0.858	0.995	

**Table 10:** The prescription errors.

Prescription Errors	Medication Underdosed	Medication Overdosed	Wrong Diagnostic Code
Out of total (%)	2.3%	3.1%	2.8%
Arad (%)	2.4%	2.4%	6.7%
Timis(%)	2.2%	3.2%	1.2%
p ^sig^ (Chi^2^)	0.884 ^is^	0.787 ^is^	<0.001 ^s^

Notes: ^is^—insignificant difference; ^s^—significant difference; ^sig^—significant difference. Bold highlights the most encountered diagnostic codes.

**Table 11:** Example of overdosed medication found.

Overdosed Medication	AR Urban	TM Urban
Amlodipine 10 mg *	X	
Lisinopril 20 mg *	X	
Enalapril 20 mg *		X
Lercanidipine 20 mg *		X
Fosinopril 20 mg *		X
Ramipril 10 mg *		X
Perindopril 10 mg *		X
Proton pump inhibitors 80 mg/day		X
Noliprel 5 mg/1.25 mg *		X
Amlodipine/Valsartan 10 mg/160 mg *		X

Notes: * 2 administrations/day.

**Table 12:** Example of underdosed medication found.

Underdosed Medication	AR Urban	TM Urban
Mebeverine (200 mg/day)	X	
Pentoxifylline (400 mg/day)	X	
Trimetazidine (35 mg/day)		X
Omega-3 acids *		X
Sulodexide *		X
Pramiracetam *		X
Nitrates *		X

Notes: * 1 administration/day.

**Table 13:** Example of wrong diagnostic code found.

Wrong Diagnostic Code	AR Urban	TM Urban
Proton pump inhibitors for arterial hypertension	X	
Dihydropyridine for dyslipidaemia	X	X
Proton pump inhibitors for cardiomyopathy	X	
Ivabradine for arterial hypertension	X	X
Strontium ranelate for arterial hypertension	X	
Digoxin and amiodarone for cardiovascular diseases	X	
Allopurinol for dyslipidaemia		X
Diosmin for arterial hypertension		X
Pentoxifylline for arterial hypertension		X
Vinpocetine for arterial hypertension		X
NSAIDs and proton pump inhibitors for cardiomyopathy		X

**Table 14:** STOPP criteria: medication with no evidence-based indication.

Medication with No Evidence-Based Clinical Indication	AR Urban	TM Urban
Proton pump inhibitors for arterial hypertension	X	
Dihydropyridine for dyslipidaemia		X
Proton pump inhibitors for cardiomyopathy	X	
Ivabradine for arterial hypertension		X
Strontium ranelate for arterial hypertension	X	
Proton pump inhibitors for arthrosis		X
Antifungals in asthma		X
Statins for hyperkinetic disorders		X
Gabapentin for cerebrovascular diseases		X
Vinpocetine for arterial hypertension		X
Neuroleptics for depressive episode		X
NSAIDs and proton pump inhibitors for cardiomyopathy	X	

**Table 15:** STOPP criteria: exceeded duration of treatment.

Medication with Exceeded Duration	AR Urban	TM Urban
H2 receptor antagonist		X
Proton pump inhibitors		X
NSAIDs	X	X

**Table 16:** STOPP criteria: duplicate drug class prescription or drugs.

Duplicated Medication	AR Urban	TM Urban
Beta2-blockers	X	
Dihydropyridines		X
Diuretics		X
Calcium channel blockers		X
Timolol		X

## Data Availability

Additional data are available from the corresponding author upon reasonable request.

## Abbreviations

ACE	angiotensin-converting enzyme
ACS	acute coronary syndrome
AR	Arad
ARB	angiotensin receptor blocker
CID	personal insurance code
COPD	chronic obstructive pulmonary disease
CV	cardiovascular
CVD	cardiovascular disease
EMPCC	electronic medical prescription for chronic conditions
eNOS	endothelial nitric oxide synthase
EU	European Union
FPP	farnesylpyrophosphate
GGPP	geranylgeranylpyrophosphate
GINA	Global Initiative for Asthma
GOLD	Global Initiative for Chronic Obstructive Lung Disease
HDL-C	high-density lipoprotein cholesterol
ICS	inhaled corticosteroid
LABA	long-acting beta2-agonist
LAMA	long-acting muscarinic antagonist
LDL-C	low-density lipoprotein cholesterol
NHIH	National Health Insurance House
NO	nitric oxide
NSAID	non-steroidal anti-inflammatory drug
OTC	over-the-counter medication
oxLDL	oxidised low-density lipoprotein cholesterol
PIM	potentially inappropriate medication
PIP	potentially inappropriate prescribing
PPI	proton pump inhibitor
PPO	potential prescribing omission
ROS	reactive oxygen species
SABA	short-acting beta2-agonist
SABINA	Short-acting beta2-agonist Use IN Asthma
SD	standard deviation
SNRI	serotonin and norepinephrine reuptake inhibitors
SPSS	Statistical Package for Social Sciences
SSRI	selective serotonin reuptake inhibitors
START	Screening Tool to Alert to Right Treatment
STOPP	Screening Tool of Older Persons’ Prescriptions
TM	Timis
WHO	World Health Organization
